# Multidimensional overview of neurofilament light chain contribution to comprehensively understanding multiple sclerosis

**DOI:** 10.3389/fimmu.2022.912005

**Published:** 2022-07-28

**Authors:** Rodolfo A. Kölliker Frers, Matilde Otero-Losada, Tamara Kobiec, Lucas D. Udovin, María Laura Aon Bertolino, María I. Herrera, Francisco Capani

**Affiliations:** ^1^ Centro de Altos Estudios en Ciencias Humanas y de la Salud, Universidad Abierta Interamericana, Consejo Nacional de Investigaciones Científicas y Técnicas (CAECIHS. UAI-CONICET), Buenos Aires, Argentina; ^2^ Unidad de Parasitología, Hospital J. M. Ramos Mejía, Buenos Aires, Argentina; ^3^ Centro de Investigaciones en Psicología y Psicopedagogía (CIPP), Facultad de Psicología y Psicopedagogía, Pontificia Universidad Católica Argentina (UCA), Buenos Aires, Argentina; ^4^ Departamento de Biología, Universidad Argentina John Kennedy (UAJK), Buenos Aires, Argentina

**Keywords:** multiple sclerosis, neurofilaments (NFs), axonal damage, diagnosis, monitoring, serum detection

## Abstract

Multiple sclerosis (MS) is an inflammatory neurodegenerative disease characterized by demyelination, progressive axonal loss, and varying clinical presentations. Axonal damage associated with the inflammatory process causes neurofilaments, the major neuron structural proteins, to be released into the extracellular space, reaching the cerebrospinal fluid (CSF) and the peripheral blood. Methodological advances in neurofilaments’ serological detection and imaging technology, along with many clinical and therapeutic studies in the last years, have deepened our understanding of MS immunopathogenesis. This review examines the use of light chain neurofilaments (NFLs) as peripheral MS biomarkers in light of the current clinical and therapeutic evidence, MS immunopathology, and technological advances in diagnostic tools. It aims to highlight NFL multidimensional value as a reliable MS biomarker with a diagnostic-prognostic profile while improving our comprehension of inflammatory neurodegenerative processes, mainly RRMS, the most frequent clinical presentation of MS.

## Introduction

Multiple sclerosis is a neurological disease characterized by CNS inflammation and neurodegeneration, demyelination, and progressive axonal loss, presenting in varied clinical forms. Alternating flare-ups and remissions progress to irreversible deterioration, eventually.

Neurofilaments (NFs) are the major constitutive proteins of the axonal cytoskeleton. These heteropolymers are classified into three types according to their size: light chain (NFL, 61.5 kDa, 543 amino acids), medium (NFM, 102.5 kDa, 916 amino acids), and heavy chain (NFH, 111.9 kDa, 1020 amino acids) neurofilaments (1). Axonal damage after pathological processes or trauma causes neurofilaments’ leak into the extracellular space (2), whence they diffuse into the cerebrospinal fluid (CSF) and reach the peripheral blood (PB). Their concentration in body fluids can be used to assess axonal damage. Many studies have reported the potential value of CSF and peripheral blood to quantify NFL as a biomarker in a variety of diseases characterized by axonal loss, like stroke, small vessel disease, HIV infection, head trauma, amyotrophic lateral sclerosis, Alzheimer’s disease, Huntington’s disease, acute spinal cord injury, neuromyelitis optica, and MS (3, 4).

In the beginning, NFL quantification was restricted to CSF, limiting its approval. Upon emerging ELISA to measure NFL in peripheral blood, reports started appearing on NFL biomarker potential (5–9). Today, further methodological advances allow reliable NFL quantification (10).

The value of neurofilaments as biomarkers of neurodegeneration, and inflammation, the advances in the knowledge of MS immunopathogenesis (11), and MRI scans correlation with damage, and clinical fluctuations are well-documented in MS (12, 13). A biomarker must fulfill certain requirements. It must show baseline levels with predictive value, clinical relevance, correlation with disease severity fluctuations, and sensitivity to therapeutic interventions (14, 15). In MS, better yet, if circulating level correlates with MRI findings.

This review examines the evidence supporting sNFL value as a biomarker in MS diagnosis, prognosis, and follow-up, portraying the current scenario of serum neurofilaments’ situation in the dynamics of collective advances in MS knowledge. It also ponders how interpreting sNFL levels with advanced MRI scans may shed light on neurodegenerative and inflammatory events and contribute to upgrading the knowledge of pathogenic immune response.

## Immunopathology and inflammation

**Figure 1:**
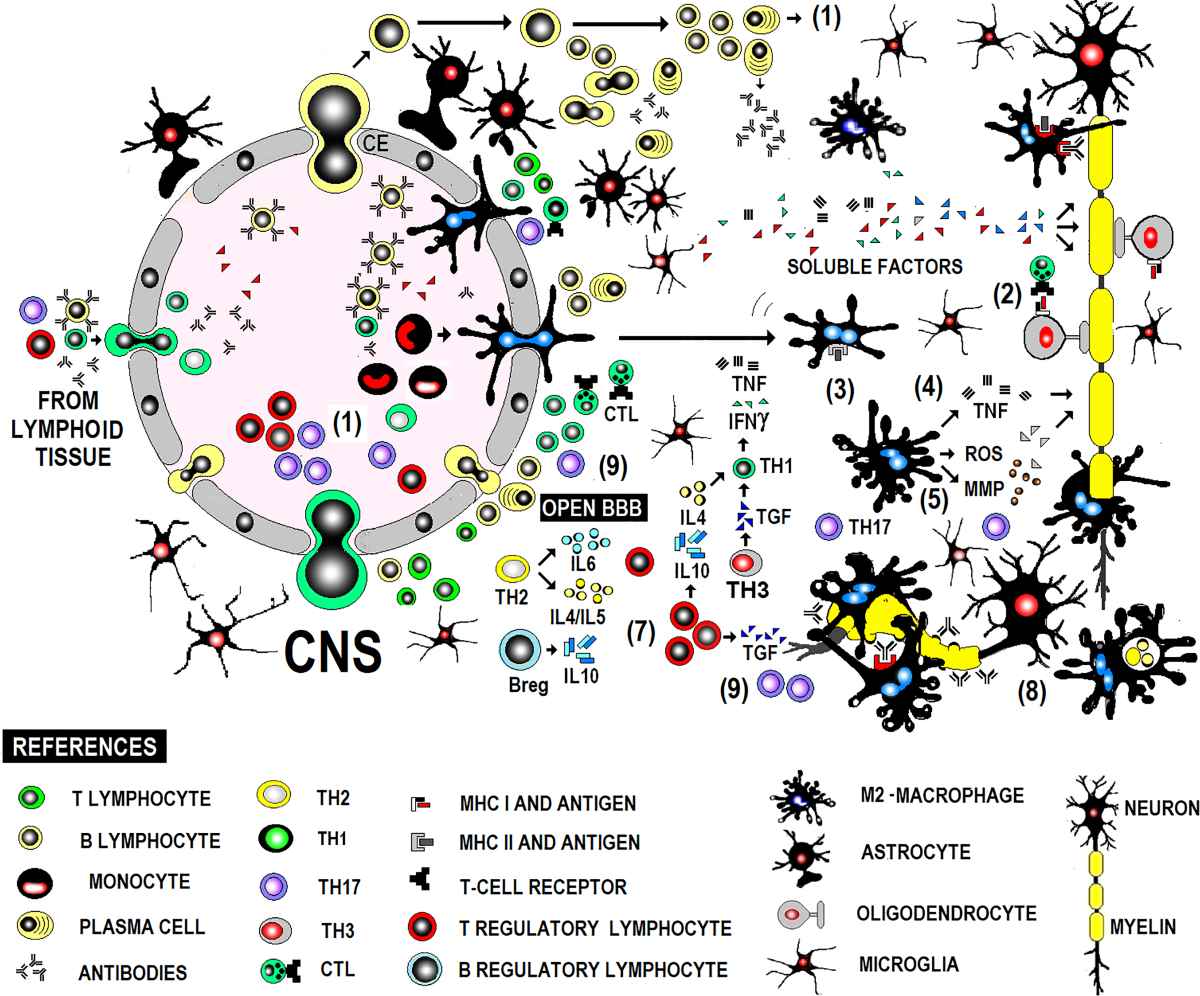
Immunopathogenesis of MS in brief.

MS is a chronic inflammatory disease of the CNS, presumably autoimmune, manifesting in genetically predisposed people. Although the environmental trigger is unknown, the autoreactive immune response is directed against CNS autoantigens due to T-cell self-antigen tolerance loss during thymic clonal deletion. Pathogenic inflammatory immune response in MS comprises T, B, and myeloid cells, acting in concert to amplify or dampen pathogenic immune responses according to activation states and the micro-milieu **(** **Figure 1** **).** MS immunopathology is given by an imbalance between pro-inflammatory immune cells and a defective regulatory immune cell pool in the periphery. An imbalance between pro-inflammatory immune cells and a defective regulatory immune cell pool in the periphery shape MS immunopathology. Immune cells phenotype-switch causes a decrease in suppressor-cells and increased infiltration of autoreactive adaptive immune cells into the CNS. Recent studies help to discriminate whether the beneficial effects of disease-modifying therapies may be related to direct effects on B and T cells or to their side-effects on antigen-presenting cells as monocytes/macrophages (11).

RRMS is the most frequent clinical MS form, presenting as a chronic evolutionary clinical profile with flare-ups and remissions, demyelination, gliosis, axonal damage, and inflammation. Despite intensive research and advances, the underlying mechanism that drives chronic demyelination is not fully understood. Although the pathogenic immune response has traditionally involved T lymphocytes and myeloid cells (macrophages), B lymphocytes play a role in pro- and anti-inflammatory responses. This triad of immune cells crossing the blood-brain barrier and causing damage to myelin and oligodendrocytes *in situ* poses the prime target for disease-modifying treatments (DMTs) in RRMS in particular. Resident cells (astrocytes and microglia) participate in both inflammatory and anti-inflammatory phases (11).

### T Cells in MS immunopathology

The experimental autoimmune encephalomyelitis (EAE) model contributed to deeming MS as a T-lymphocyte-mediated disease (16). Further studies implicated closely related genes like those codifying for antigen-presentation, T-dependent pro and anti-inflammatory effector response, and thymic T lymphocyte differentiation of pathogenic T lymphocytes. From an effector immune response perspective, major histocompatibility complex (MHC) alleles can increase or decrease predisposition to the disease. Some of such respectively predisposing and protective alleles are related to antigenic presentation to autoreactive helper TCD4 lymphocytes of myelin-derived proteins acting as autoantigens as the proteolipid protein (PLP), myelin basic protein, and myelin oligodendrocyte glycoprotein (MOG) (17).

Traditionally, models of RRMS pathogenesis were based on the central role of inflammatory Th1 lymphocytes and anti-inflammatory Th2 helper lymphocytes (counter-regulation) after the IFNγ findings in MS lesions. However, Th1 lymphocytes do not act alone. The circulating level of IL-17-producing Th17 cells is associated with more severe disease forms, presenting active lesions (18, 19).

Th17 cells can switch to a Th1-like phenotype, which releases IFN-γ and IL-17, detectable in brain tissue, CSF, and peripheral circulation of RRMS patients in the relapse phase (20–22).

Th3 cells were first described in the experimental autoimmune encephalitis (EAE) mouse model and later in humans as CD4+FOXP3+ regulatory T cells. Unlike the well-characterized T regulatory (Treg) cells, Th3 cells do not express transcription factor FOXP3. Further findings suggest that Th3 cells are a different lineage from natural Treg CD25+CD4+ Treg cells. Whether Th3 cells are induced Treg cells is unclear in the lack of a specific marker. Th3 cells secrete anti-inflammatory cytokine transforming growth factor-beta (TGF-β) and inhibit Th1 and Th2 cells (23).

Treg cells partly counteract effector T lymphocyte activity (24). Yet, this has not been observed in the clinical variant of RRMS with Treg cells specific for myelin oligodendrocyte glycoprotein (MOG) (25). Treg blood level in RRMS patients compares to that in healthy controls., while Treg suppressive activity is lower in the former (26). Various approved disease-modifying therapies indirectly modulate Treg cells. Glatiramer acetate, the first treatment line for RRMS, increases Treg circulating levels (27), while IFN-β increases the number of Treg cells with a CD4+, CD25+, and Foxp3+ phenotype (28).

The anti-CD25 monoclonal antibody daclizumab interferes with activated T lymphocyte proliferation, not affecting cells that express the low-affinity receptor for the IL-2 heterodimer receptor, which does not express the gamma subunit of the high-affinity receptor for natural killer cells (29). This might help to understand why a therapy targeted on the TCD4 strain is insufficient to interfere with the pathogenesis of the disease. The lesion’s cellular infiltrate is mainly made up of TCD8 + cells. CD8 + T cells secrete IL-17 and cause Tc17, increased in active RRMS lesions (30). The evidence supports an immunopathogenic model based on the imbalance between proinflammatory Th1, Th17, and Tc17 lymphocytes, on the one hand, and peripheral Treg lymphocytes, on the other. At different stages of the immune response, effector and regulatory T cells interact with different antigen-presenting cells as B lymphocytes, macrophages, dendritic cells, and microglia.

Lymphocytes are largely perivascular and may be involved in inducing tissue damage or have a regulatory function depending on the stage of the injury. This distribution of T and B lymphocytes, distant from the sites of neurodegeneration and demyelination, has been described in both acute and chronic active lesions and suggests that their participation in inflammation and neurodegeneration is mediated in part by soluble factors and interaction with microglia and macrophages (31). Acute demyelinating lesions have activated M2 macrophages and may become chronic, showing positive iron labeling usually in the microglia/macrophages at the edge of the lesion, typically expressing M1 activation markers, with perivascular lymphocytic infiltrate both cases **(** **Figure 1** **).**

The triggering mechanism and the antigenic specificity of infiltrating T and B cells are uncertain. Activated lymphocytes differentiated to Th1, Th17 proinflammatory effector lymphocytes or Treg, Th2 or Th3 anti-inflammatory effector lymphocytes are recruited centrally (1). They stimulate pro or anti-inflammatory effector mechanisms indistinctly during flare-ups and remissions. T cell stimulation can be induced by the interaction with B cells or myeloid cells. B cells also differentiate into plasma cells, affecting the immune response *via* antibody secretion. Autoantibodies contribute to CNS inflammation *via* CNS antigens opsonization and complement fixation, while B cell cytokines directly affect myeloid cells, inducing pro-inflammatory or anti-inflammatory phenotypes. Autoreactive immune responses are suppressed *via* different mechanisms, including IL-10 secreted by Treg cells, Breg cells, and anti-inflammatory myeloid cells, maintaining a balance between pro- and anti-inflammatory immune cells. Proinflammatory lymphocytes activate macrophages in a T-dependent manner. Macrophages, glial cells, mainly oligodendrocytes (2), and B lymphocytes actively participate in antigen presentation to effector lymphocytes. Lymphocytes, glial cells, and macrophages release TNF and lymphotoxin, causing tissue damage. IFNγ enhances the afferent mechanisms of the immune response by stimulating the expression of MHC (3). Macrophages produce mediators (MMP, ROS) that amplify damage (4, 5) and attack oligodendrocytes and myelin *via* TNFα-mediated cytotoxicity. B cells differentiate into antibody-secreting plasma cells. Antibodies contribute to added antibody-mediated cellular cytotoxicity (ADCC) and complement-mediated mechanisms (6). Regarding immunophenotype of the cellular infiltrate, the dominant inflammatory cells are activated CD8+ T lymphocytes (cytotoxic T lymphocytes) in early stages of active lesions during the acute phase and a phenotype of memory cells resident in tissue with focal activation in lesions with ongoing demyelination and neurodegeneration during the progressive stage. Disease remission is associated with increased production of TGFβ, which regulates the effector mechanisms mentioned above (7), and less inflammatory activity of myeloid cells. The shift from a pro-inflammatory to anti-inflammatory condition includes Breg cells that modulate T-cell and myeloid cell functions, secreting anti-inflammatory cytokines. Myelin phagocytosis by macrophages (8) might have an anti-inflammatory role and is documented by electron microscopy. Pathogenic Th1 lymphocytes are believed to be produced by IL23-commanded Th17 populations (9) but not by IL12-commanded Th1 populations in the clinical form with anti-MOG antibodies present and damage produced by antibodies and the complement. CD20 + B cells may be largely found in perivascular inflammatory aggregates associated with the activity of the lesion at any stage of the disease. Demyelination and neurodegeneration occur remote from T and B lymphocytes, critically associated with activated microglia and macrophages, whose deleterious action would be mediated by soluble factor (s) of lymphocytic origin.

### Myeloid cells in MS immunopathology

Since the initial models of experimental EAE, the central role of myeloid cells, in particular, monocytes and macrophages in the cell infiltrate, has been sustained in MS.

Axonal degeneration is associated with the inflammatory activity of activated macrophages in acute active lesions and microglia in active and latent chronic lesions, despite demyelination presents a variable course in MS (RRMS and PMS) and correlates with NFL serum level (13, 32).

Monocytes may secrete IL-6, IL-12, TNF-α, and IL-10. Untreated MS patients show high monocyte IL-6 and IL-12 levels compared with treated or healthy controls (33) that fluctuate according to disease progression (34). However, careful interpretation is required due to the phenotypic plasticity of this circulating myeloid lineage. Phenotypic heterogeneity of circulating monocytes is classified according to the relative expression of CD14, the putative receptor for LPS, and CD16, known as IgG Fc fraction type III receptor (FcγRIII). Traditional monocytes show a CD14++ CD16− phenotype, non-traditional monocytes show a CD14 ++ CD16 + and an intermediate CD14+ CD16++ phenotype (35), all differentially contributing to MS immunopathology. CD16 + monocytes promote T cell entry into the CNS through the BBB (36), and their circulating levels are higher in MS patients compared with healthy controls and are modified by DMTs (37, 38). Infiltrating monocytes/macrophages and resident microglia phenotypes may, due to phenotypic plasticity, take on an anti-inflammatory (neuroprotective) or inflammatory (neurodegenerative and demyelinating) role. An intermediate-activation phenotype has also been suggested (39). Activation status and phenotypic behavior have been related to the antigen-presenting activity of MHC II-associated autoreactive antigens in both microglia and macrophages. Phagocytosis, however, is not only associated with antigenic presentation and deleterious activity (40) but gives rise to the reparative, anti-inflammatory, and remyelination-inducing phase through waste elimination, as well (41).

Monocytes isolated from MS patients treated with glatiramer acetate and fingolimod showed a less pro-inflammatory and a more anti-inflammatory phenotype. Glatiramer treatment increased IL-10 and TGF-β secretion and decreased TNFα, IL-12, and IL-1β levels (42–44).

Further studies on phenotypic heterogeneity, secretory profile, and associated disease phase are required to better understand myeloid cell participation in pro- and anti-inflammatory processes and design new specific therapies.

### B Cells in MS immunopathology

Autoreactive B lymphocytes are under central and peripheral control to keep self-tolerance. Treg cells participate in peripheral tolerance of autoreactive B lymphocytes, and loss of tolerance due to their deficient suppressive capacity has been described in MS patients (45).

When tolerance against self-antigens is lost, autoreactive clones appear due to unknown triggers. Self-reactive memory B cells produced in MS patients’ spleen and lymph nodes can act as effective APCs (even more than typical APCs), binding specific autoantigens to their B-cell receptor site and presenting them *via* MHC to CNS-specific pathogenic T cells. Pathogenic T effector cells include T helper-17 (Th17), T helper-1 (Th1), and Treg and can be activated, modulating disease activity in RRMS phases. Mechanisms other than antibodies pertaining to T-B interaction may influence MS pathogenesis. Typically, the autoantigen is internalized, processed, and presented by memory B cells associated with MHC II to CD4 + T lymphocytes (46) in the presence of costimulatory signals (CD40, CD80, and CD86) expressed on the surface of the B lymphocyte (47).

B cells also contribute to disease pathology with antibody-dependent and independent effects. First, their participation in MS was only related to TB lymphocyte collaboration, activation of B lymphocytes, and differentiation into plasma cells producing autoantibodies, visualized as oligoclonal bands (OCB) in CSF of MS patients. OCBs result from high amounts of IgG, found in over 90% MS patients, and IgM, found in 30-40% of MS patients, produced by B cells differentiated into plasma cells and represent a hallmark in MS diagnosis (48, 49). However, OCB-derived antibodies may have a more heterogeneous origin than considered. Within the CNS, antibodies trigger complement activation and demyelination. Although antibodies are directed against CNS constituents like MOG, MBP, neurofilaments (neurons), astrocyte antigens, and proteins, they may also be found in healthy individuals (50, 51). Then, OCB antibodies would be specific for endogenous cellular debris proteins (11, 52). In RRMS, both the death of oligodendrocytes and neurons and demyelination are associated with soluble components of B lymphocytes, and the removal of antibodies does not interfere with damage associated with B lymphocytes. Hence, pathogenicity would be mediated by cytokines, independent from antibodies (53, 54).

At present, the pathogenic role of autoantibodies remains controversial due to the lack of consensus. The anti-inflammatory efficacy of anti-CD20 therapy in RRMS patients supports B lymphocytes’ immunopathogenic role (55, 56) attributed to the interference on antigen-presentation to T lymphocytes. Blocking lymphocyte response (via reduction of IFNγ) and its effect on the macrophage (reducing IL-6 and TNFα), anti-CD20 favors secretion of the anti-inflammatory IL-10, IL-35, and TG F-β derived from T lymphocyte-macrophage interaction (11). Additionally, anti-CD20 therapy is associated with a decrease in granulocyte-macrophage colony-stimulating factor (GM-CSF), an inflammatory cytokine. Briefly, anti-CD20 antibodies not only interfere with T-B interaction but also affect CD20-expressing T lymphocytes like the T-CD4 and T-CD8+ cells, myelin specific (57).

The evidence supports the immunophenotype of the cellular infiltrate. Early stages of active lesions in the acute phase typically show activated cytotoxic CD8+ T lymphocytes. In focal CNS active lesions with ongoing demyelination and neurodegeneration during the progressive stage, resident memory cells are the dominant phenotype. At any stage of the disease, CD20+ B cells may be largely found in perivascular inflammatory aggregates related to lesion activity.

## Basic structural aspects of neurofilaments

Since in 1896 Rudolph Albert von Kölliker (^1^*) coined the term axon to describe the long slender cables that transmit signals away from cell bodies, remarkable advances have been made in the physiology and pathophysiology of the nervous system.

Here, we describe NFs’ essential structural aspects in the axonal cytoskeleton to highlight their relevance in neurodegeneration and diagnostic potential in neurodegenerative diseases.

The axonal cytoskeleton is a finely regulated system that supports and maintains structural axon integrity. Unique structures like actin microfilaments (6 nm diameter), microtubules (20 to 25 nm diameter), and neurofilaments (10 nm diameter) assembled together keep axonal shape and size, nutrients’ transport, and organelles. They also delimit specialized membrane domains and regulate axonal growth and focal adhesions (58).

Neurofilaments are neuron-specific and the major structural proteins in neurons, standing for over 85% of total protein content. They are synthesized in the neuronal soma and concentrate in axons, particularly large ones (59). Axonal damage causes neurofilament leakage into the CSF (60). Extracellular detection of NFL is a sign of neuronal damage (61) due to neuron-specificity (60). Once synthesized, NFs undergo post-translational glycosylation and phosphorylation. Phosphorylation varies with the axonal type and nerve tract and is almost negligible in small-caliber axons (62).

## Neurofilament light chain quantification assays

The quantification of specific, sensitive, and reliable axonal degeneration biomarkers in CSF and blood offers an important advance in monitoring MS disease activity.

At first, immunoenzymatic assays like ELISA, Western Blot, and Dot Blot were developed to detect NFs in CSF and peripheral blood. In time, first-generation (immunoblotting), second-generation (enzyme-linked immunosorbent assay) with limited sensitivity, third-generation (electrochemiluminescence), and fourth-generation assays (single-molecule array) were designed. Today, reliable NFL determination in the blood concentration range is available (10). The ultra-sensitive single molecule arrays fourth-generation technique shows a 126- and 25-fold blood NFL detection sensitivity compared with conventional ELISA and ECL-based assays, respectively (6). Serum NFL level, nearly 50-100 times lower than CSF’s and yet, closely related (10), can be used as a marker of brain damage (3, 63).

## Circulating anti-neurofilament antibodies

**Figure 2:**
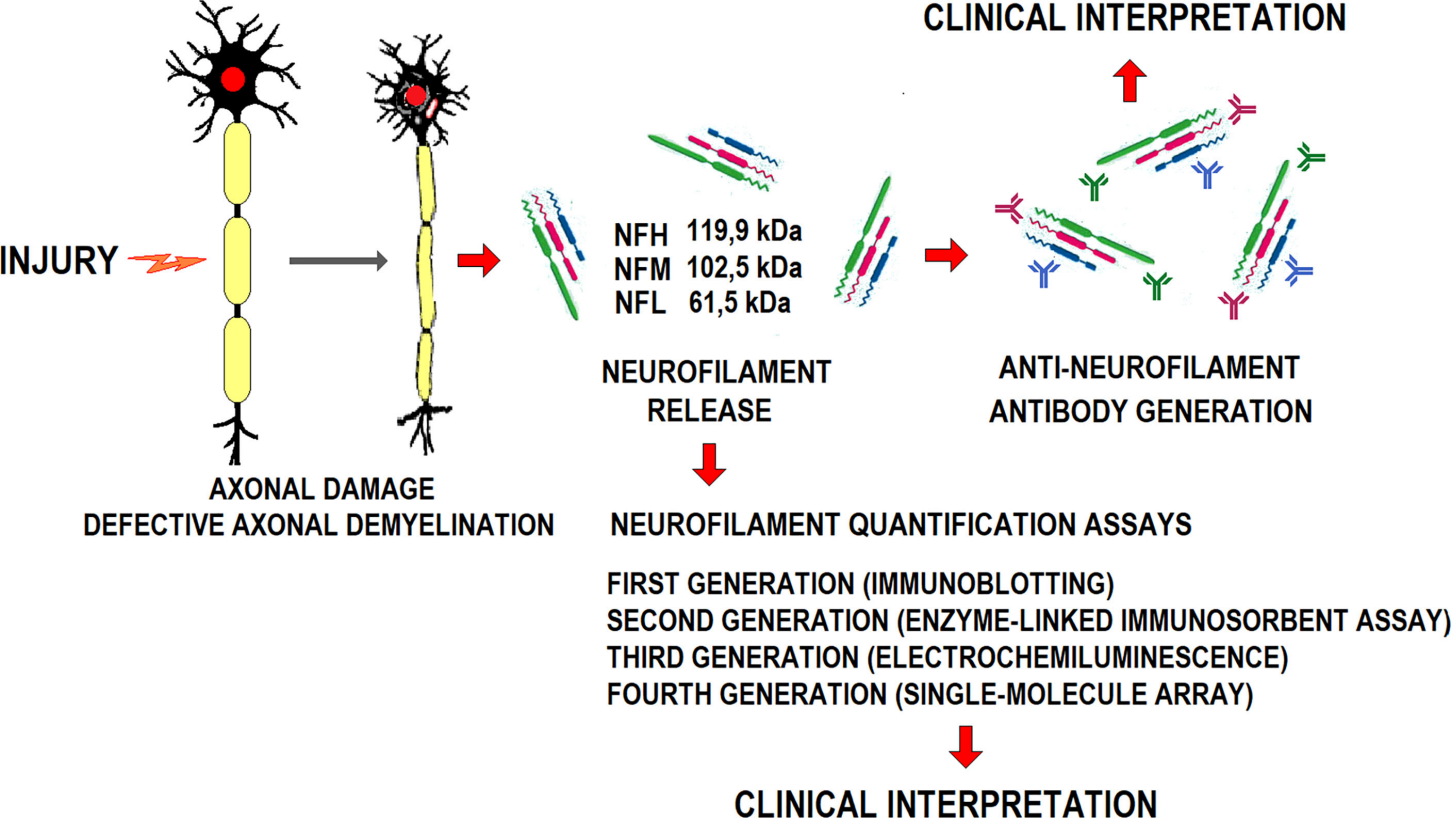
Schematic representation of axonal damage generation, the release of constitutive neurofilaments, anti-NFL antibodies generation, and their diagnostic potential.

Damaged axons release neurofilaments to the CSF. Once in the peripheral blood, these highly immunogenic proteins induce a specific humoral response (64) **(** **Figure 2** **).** Anti-neurofilament antibodies’ blood level varies with neurological disease progression. However, neither their pathogenicity nor potential value in monitoring disease progression is clear.

Fialová et al. (65) reported an association between the CSF/blood anti-NFL level ratio and a higher risk of clinically isolated syndrome (CIS) conversion to MS. A later study failed to find a relationship between serum and CSF anti-NFL concentrations (66).

Anti-NFL levels correlated with disease duration and EDSS score (67) and decreased with natalizumab treatment (68). Anti-NFL antibodies are cleared from circulation when circulating NFL levels decline (68). Yet, the evidence on the clinical utility of circulating anti-NFL antibodies is not conclusive. The current interpretation of circulatory anti-neurofilament antibodies is uncertain. Whether they may interfere with circulating neurofilament determination or MS clinical assessment and monitoring is uncertain. Since their circulating levels correlate with NFL decrease in disease remission, they are likely eliminated by immune system-mediated clearance mechanisms. There is a need to clarify this issue in future research to draw solid conclusions about the impact of anti-NFL antibodies on circulating NFL and the clinical interpretation.

## Clinical value of NFL as a biomarker in multiple sclerosis

Multiple sclerosis diagnosis is based on McDonald’s diagnostic criteria. These link clinical manifestations with typical lesions in magnetic resonance images (MRI) (69, 70), CSF analysis, and visual evoked potentials. Finding reliable laboratory techniques to diagnose this complex disease is still a challenge.

Oligoclonal bands (OCB) are detected in the CSF of nearly 95% of MS patients, conveying a highly relevant diagnostic contribution (71). Their finding is a powerful predictor of CIS conversion to clinically diagnosed MS, regardless of the MRI lesion load (72). Either a high NFL level or the presence of OCB announces a fast CIS progression to MS (73).

The complexity of this disease, with an unpredictable clinical course, demands sensitive and specific diagnostic, prognostic, and therapy monitoring follow-up indicators (74). A biological marker has to be sensitive to health and disease fluctuations and to therapeutic — pharmacological or other — interventions. The ideal biomarker must show good replication, and be sensitive, specific, time stable, and cost-effective.

In MS, finding suitable biomarkers faces several methodological problems. Partial knowledge of MS pathophysiology, the large fluctuation in the concentration of marker-like substances in blood and urine, the variability of immunological parameters unrelated to the disease, and the synergistic or antagonistic effect of certain biomarkers, are some of them.

Many studies have evaluated potential biomarkers of neurological disease in body fluids. Promising candidates for early diagnosis, reliable prognosis, and therapeutic response monitoring have been identified. So far, the evidence suggests that axonal damage causes neurofilaments’ leakage to the surrounding extracellular space, some stable enough to be detected by specific tests in CSF (75) and blood samples (10, 76, 77).

Will these putative markers prove reliable to evaluate axonal degeneration in MS, they might help predict and monitor disease fluctuations and therapeutic efficacy (63).

### Clinical evidence based on NFL quantification in CSF of MS patients

Lycke et al. (78) developed an NFL-specific homemade ELISA method, using a purified chicken antibody to test the potential use of NFs protein subunits as surrogate markers of axonal degeneration in MS. The cerebrospinal concentration of NFL was measured in 60 patients with clinically diagnosed RRMS. Samples were collected at disease onset and 2 years later. The results showed a 78% increase in CSF concentration of NFL with time, associated with disability. Likewise, a few years later, a cross-sectional-observational study including 66 MS patients and 50 healthy controls showed that CSF concentration of NFL increased during relapse (79).

A 6-12-month longitudinal observational study performed with 83 RRMS patients, 9 SPMS patients, and 28 healthy controls reported that CSF concentration of NFL decreased to a lesser extent in SPMS after natalizumab treatment compared with RRMS (80).

More recently, NFL concentration in CSF at the time of diagnosis was retrospectively analyzed in 99 clinically diagnosed MS patients to assess whether it could be used to predict MS progression. Out of the total, 94 complete records, including data at the time of diagnosis, and disease severity at 5 and 14 years, were retrieved. A high level of NFL was associated with a 3-fold increase in MS severity risk, according to bivariate and multivariate logistic regression analysis estimates, particularly in recently relapsing RRMS patients. Besides, around 60% of patients with high CSF NFL level (> 386 ng/mL) progressed from RRMS to SPMS over a 14-year follow-up compared with 30% of patients with moderate or low level (<386 ng/mL). These studies suggest that, early in the disease, CSF NFL levels might anticipate disease progression. Likewise, a high CSF NFL level might suggest conversion to the progressive disease. A higher CSF NFL level has been found in clinical isolated syndromes, progressing to RRMS within 3 years, compared with those that do not (77). Cerebrospinal NFL concentration is high in MS patients with cognitive impairment (81).

### Clinical evidence based on NFL quantification in peripheral blood of MS patients

Different studies have reported unequivocal evidence supporting the role of NFL as a biomarker of MS. One MS cohort, including CIS, RRMS, PPMS, and SPMS patients, had higher sNFL levels compared with controls (5). Serum NFL concentration was considered a reference marker linking CIS to MS (66). Patients with MS have a high sNFL level (9, 82).

In January 2019, the International Progressive MS Alliance examined data of NFL in serum and plasma from both relapsing MS and progressive MS. The panel concluded sNFL was a plausible marker of neurodegeneration, measurable with acceptable accuracy, sensitivity, and reproducibility, but standard procedures for sample processing and analysis should be established (83).

Circulating NFL has been proposed as a biomarker of acute and chronic neuronal damage in early MS (8). A decrease in sNFL level was observed in a clinical cohort of 286 MS patients undergoing DMT. Whether decreased sNFL levels predict long-term outcomes is still uncertain (63).

MS patients in a clinical relapse or with radiological activity had higher sNFL levels than those in remission or without new MRI lesions (8, 63).

The decrease in sNFL level was correlated with improved EDSS scores and neuropsychological outcome and brain volume changes 12 and 24 months later. Recent studies show that NFL blood concentration makes up a robust MS biomarker (74).

In sum, NFL is more than a non-specific axonal damage marker like C-reactive protein, released in response to central or peripheral nervous system damage. Serum NFL levels are very high in MS patients with strong disease activity and predict poor outcomes. Next, a biomarker validation platform is required to speed up biomarker identification in MS and other neurological conditions and other comorbidities (83). So far, the evidence suggests the potential applicability of sNFL as a routine tool in the general practice of neurology, aiding in prognosis, as a marker of response to treatment, and as a treatment target endpoint. Further studies to find reliable cut-off values with contingent comorbidities will contribute to more customized medical practice (83). **Table 1** summarizes recent evidence of sNFL as an MS biomarker.

**Table 1:** Findings supporting the biomarker value of circulating NFL concentration in MS.

Reference #	Results	Value
(63)	NFL level was higher in patients with either RRMS (16.9 ng/L) or PPMS or SPMS (23 ng/L) than in controls (10.5 ng/L).	diagnostic
(84)	NFL level was associated with gadolinium-binding T1 lesions up to 2 months back and 1 month forth.	prognostic
(6)	NFL fluctuation correlated with EDSS score and neuropsychological outcome variation over 24 months. Brain volume decreased faster in patients with higher baseline NFL levels. The increase in NFL levels predicted the increase in brain lesions.	prognostic
(85)	Baseline NFL levels were associated with the number of gadolinium-binding lesions and the accumulation of new lesions in T2. Patients with a high rate of cerebral atrophy progression had high NFL levels.	disease activity biomarker
(86)	NFL level at the initial stages of the disease correlated with brain lesions detected ten years later, including cerebral parenchymal fraction and volume of hyperintense lesions in T2 sequences.	prognostic
(7)	Over 6.5 years’ follow-up, NFL level above the 90th percentile of control values was an independent predictor of the following year worsening EDSS in MS patients. Lesions were independently associated with increased NFL level. The higher the NFL percentile, the more pronounced were brain and spinal volume losses.	prognostic

### Clinical evidence based on NFL serum level change in response to disease-modifying treatments

The decrease in NFL level is associated with clinical and imaging outcomes. High-efficacy therapies like alemtuzumab and fingolimod treatments induced longer-lasting responses than interferon-β (63). Riluzole, as a complementary neuroprotective agent to weekly intramuscular interferon-b (IFN-b)-1a injection, did not affect sNFL level (6).

Either mitoxantrone or rituximab was associated with a decrease in NFL concentration after 12-24 months’ treatment in PPMS (87).

Treatment with natalizumab for 60 weeks was also associated with a decrease in NFL level in CSF in a single-arm, open-label prospective cohort study. Changes in NFL level in CSF correlated with clinical improvement during natalizumab or monthly methylprednisolone treatments (88).

One longitudinal, observational study evaluating 243 RRMS patients at baseline,12, and 24 months showed that plasma NFL level decreased after fingolimod treatment, even 24 months later (89).

Compared with placebo, a decrease in blood NFL concentration was observed in fingolimod and ocrelizumab trials in PPMS (INFORMS and ORATORIO, respectively) and with siponimod and natalizumab in SPMS [EXPAND and ASCEND trials, respectively] (90, 91).

A large change in NFL was observed unrelated to inflammatory activity in both the siponimod and natalizumab trials (92, 93).

In the ASCEND and INFORMS trials, a robust decrease in NFL level was observed regardless of the lack of any clinical benefit. Higher NFL blood concentration found in SPMS patients compared with PPMS patients is considered a likely predictor of brain atrophy.

On initial analysis of the SPRINT-MS phase 2 trial, ibudilast was reported to have no effect on the concentration of NFL in serum or CSF (94).

Individualized management of MS patients requires MRI (69) for therapeutic monitoring. As of the evidence, following-up NFL level in response to disease-modifying therapies (DMTs) poses potential value as a monitoring tool. **Table 2** shows studies carried out in the last seven years reporting a decrease in NFL level in response to DMTs in chronological order.

**Table 2:** Decrease in NFL level in response to disease-modifying therapies.

Reference #	DMT	QR-NFL
(88)	Natalizumab	37%
(95)	IFN or glatiramer acetate switch to rituximab	21%
(90)	Natalizumab	20%
(89)	Fingolimod	33%
(85)	Natalizumab	<16 pg/mL
(15)	Fingolimod vs IFNβ1α	38%
(94)	Ibudilast	ND

ND, No difference; QR-NFL, quantitative reduction in NFL.

### Normalization of sNFL data and clinical value

A recently published study of a Swiss MS cohort (SMSC) on the key phases in MS evolution and new treatments found that sNFL percentiles and Z-scores showed a gradual increase in the risk of acute (e.g., relapse and lesion formation) and chronic (worsening disability) disease activity. Elevated Z-scores exceeded the sNFL absolute cutoff values for diagnostic accuracy. Values of sNFL Z-score values decreased in MS patients treated with monoclonal antibodies (alemtuzumab, natalizumab, ocrelizumab, and rituximab) and, to a lesser extent, with oral therapies (dimethyl fumarate, fingolimod, siponimod, and teriflunomide). However, sNFL Z scores remained elevated for traditional treatments (interferons and glatiramer acetate). The results were fully supported in the Swedish MS Registry validation cohort (n=4341).

Taken collectively, these data show that using sNFL percentiles and Z-scores allows to identify people at risk of severe MS and suboptimal therapeutic response beyond the clinic and MRI images, and specifically those undergoing remision (96).

### Newly emerging technical platforms characteristics and advantages

Considerable evidence confirms neurofilaments, NfL in particular, as a reliable neurodegeneration biomarker. Circulating NFL level is measurable, sensitive to neurodegeneration progression, disease activity, and disability (74, 97). It also correlates with MRI lesion images (3, 6, 7, 63). NFL was first measured using an ELISA based on polyclonal antisera developed by Rosengren et al. ( (98), later upgraded to a highly specific monoclonal antibody-based assay against NFL-epitopes (99). Then, new monoclonal antibodies gave rise to a new ELISA generation (100),allowing NfL quantification in small CSF aliquots, but with low sensitivity for the less invasive peripheral NFL quantification. Considerable evidence confirms neurofilaments, NfL in particular, as a reliable neurodegeneration biomarker. Circulating NFL level is measurable, sensitive to neurodegeneration progression, disease activity, and disability (74, 97). It also correlates with MRI lesion images (3, 5, 7 63). Electrochemluminescence (ECL)-based immunoassays have recently increased blood NFL quantification sensitivity (101–103), not yet enough to detect the lowest NFL level in MS patients (101, 104).

Simoa (Single-molecule array) technology platform development, particularly suitable for ultra-sensitive protein detection in peripheral blood, represents a breakthrough in NFL quantification (81, 104). Simoa is 125 and 25 times more sensitive than conventional ELISA- and ECL-based assays, respectively. It detects as low as 0.1 pg/mL of protein (103). Over 50% and 60% of serum ELISA and ECL measurements respectively were found below detection level relative to Simoa (103). Peripheral blood NFL levels rendered by Simoa show good correlation with clinical and radiological findings (3, 63, 89) further supporting NFL reliability as an MS biomarker.

NFL values in blood and CSF samples from individuals with various neurodegenerative diseases showed a strong correlation for ECL (r: 0.78/p<0.001) and Simoa (r: 0.88/p<0.001), but weak for ELISA (r: 0.38/p<0.03) (103).

Comparable blood NFL levels were obtained by ECL and Simoa, but not by ELISA and ECL, or ELISA and Simoa (103, 105). Now, across-technologies calibrated and validated cut-off NFL values need to be determined (9, 103). Simoa is the blood neurofilament light chain (bNFL) assay of choice because of its low detection limit, simplicity, speed, and longitudinal sampling feasibility. Siemens is developing an immunoassay for bNFL using Quanterix NFL antibodies, aimed at obtaining a good correlation with Simoa bNFL level to offer a platform for routine NfL testing, speeding up NFL tests availability for patients across the world based on standard criteria. High variability between CSF and sNFL level was found in a single MS patient. NFL levels in plasma, serum, and CSF samples of the same individual, with and without brain pathology, were compared using Simoa. Serum and plasma NFL levels were strongly correlated, unlike CSF and serum or plasma levels, which were not (106).

NFL levels in CSF were reported around 200-fold higher than in plasma or serum across different populations (104, 106, 107). Even though plasma and serum levels were highly correlated, the former were systematically lower than the latter. Similar findings have been observed in neurodegeneration mice models (3, 108).

Other authors examined the effects of DMTs on CSF, serum, and plasma NFL level (89, 107), and suggested that serum levels might be more useful than plasma levels for monitoring and following-up.

The between-compartment differences in NfL levels that does not invalidate the good between-correlation may be explained by the proximity to the site of damage and the varying integrity of the blood-brain barrier, particularly prone to increased permeability upon inflammation, including concurrent peripheral inflammatory or infectious processes (107, 109, 110).

Serum NFL levels obtained by SimoaTM and EllaTM (enzyme-linked lectin assay) immunoassays were strongly correlated (ρ = 0.86, p < 0.0001) across 203 MS patients from the OFSEP (Observatoire Francais de la Sclerose Plaques) (111). Although the EllaTM instrument overestimated the values by 17%, data linearity (p = 0.57) allows a correction factor applied to the results. Ella TM-measured sNFL levels correlated with age and EDSS-score, increasing in active MS. Results suggest that SimoaTM and EllaTM assays are equivalent and can be used in routine clinical practice (111).

### Serum neurofilaments in differential diagnosis

NFLs are released in a variety of physiological and pathological conditions, making them unreliable for differential diagnosis. Longitudinal studies show that NFL level increases in MS, traumatic brain injury, and stroke over time (Mud 2020). Neurodegenerative chronic conditions like amyotrophic lateral sclerosis (ALS), Alzheimer’s, Parkinson’s, and Huntington’s diseases and traumatic brain injuries present high NFL levels as well (81, 112). Yet, specific neurofilament subunits may reflect different neurodegenerative processes (113). NFH undergoes extensive phosphorylation, influences axonal transport dynamics, and is ALS-specific (114). NFL level is high in peripheral nervous system diseases (115), limiting its use as a differential diagnosis tool.

Even when longitudinal studies are becoming appealing to the medical community, neither standardized normal cut-off values nor the optimal sampling frequency over follow-up (2, 116) have been established.

Confounding factors like age, sex, body mass index and sample size require additional studies to avoid misleading NFL data (97, 117).

Establishing optimal cut-off values is essential to using NFL as a surrogate biomarker and allow timely medical intervention. Currently, there is no consensus on cut-off values to estimate the risk of conversion from radiologically isolated syndrome (RIS)/CIS to clinically defined MS, and accurately predicting disease progression and positive response to treatment, i.e., the slightest relevant variation in NFL value. The variety of experimental designs and statistical procedures makes data comparison between studies difficult, partly contributing to the lack of consensus.

NFL cut-off levels range between 400 and 1000 ng/L in CSF (63, 81, 118) and between 3 and 30 ng/L in serum (74, 110 119, 119). Other authors use percentiles across ages (3). Once again, the lack of procedural uniformity across studies makes comparison difficult.

Further investigation should determine reliable cut-off values (97, 116, 120, 121).

In this review, we rely on the same assumptions used by Bittner et al. (116). Although the data have been simplified, they offer an approximate range of expected values in clinically relevant scenarios, yet not as validated cut-off points.

## Towards an integrated view of serial and image biomarkers

**Figure 3:**
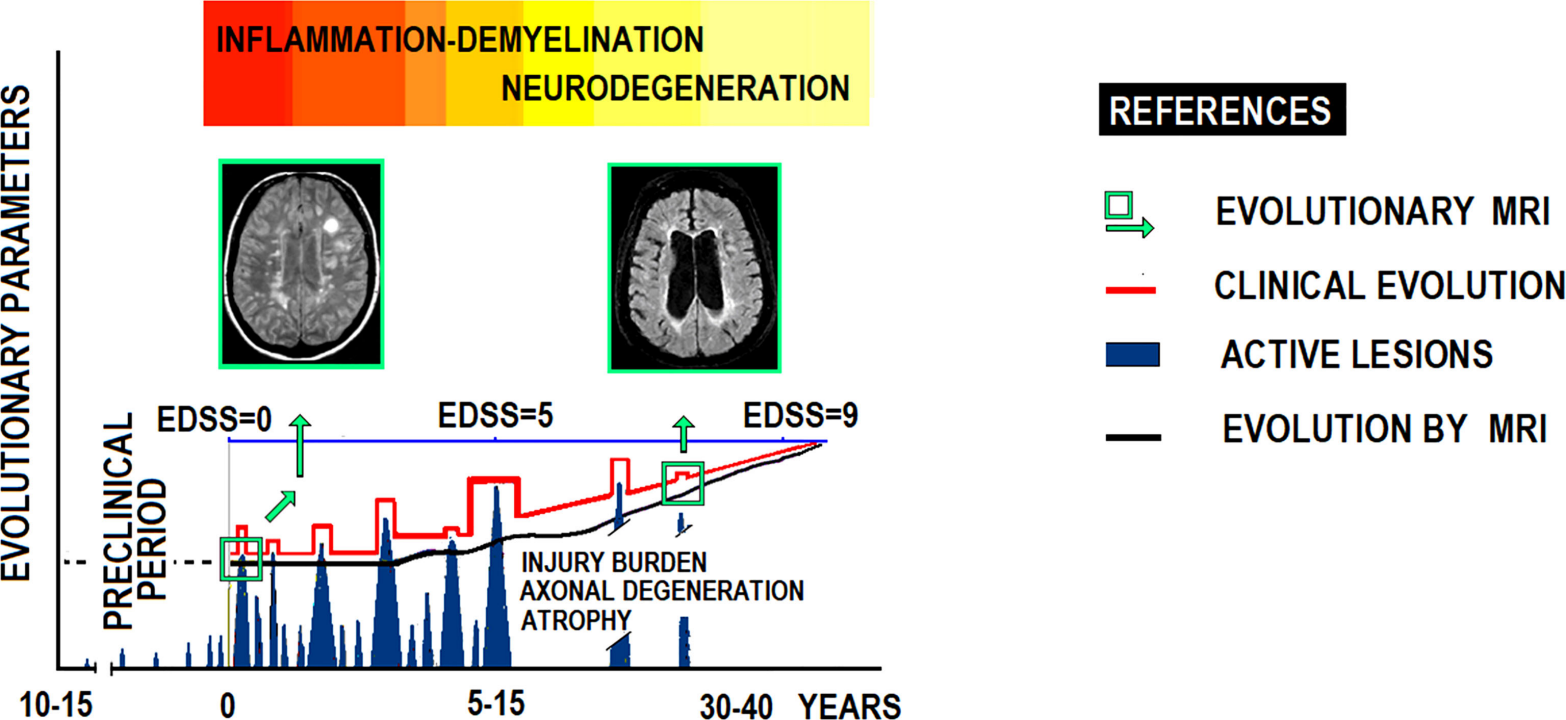
Clinicopathological evolutionary profile of RRMS.

Traditional diagnostic and evolutionary criteria for MS are mainly based on clinical relapses associated with white matter lesions (WML) on MRI scans **(** **Figure 3** **)**. However, MS misdiagnosis remains a major clinical problem.

There is a growing unmet demand for clinical research in MS to understand the clinicopathological impact of chronic CNS inflammation, a crucial target for future DMTs. To meet this, reliable biomarkers of inflammation status that integrate images and peripheral blood markers are needed.

The prevailing MS clinical form, 80-85% of cases, presents relapses and remissions with partial neurological recovery. In time, most patients enter a progressive phase of increasing disability with occasional relapses. At this stage, axonal damage seems to predominate over inflammation. This figure illustrates MS’ natural history according to clinical expression and MRI scans, showing relationships between clinical course, flare-ups, neurological disability (EDSS), lesion load (MRI), brain atrophy (brain parenchyma), and axonal damage with nuclear magnetic spectroscopy. EDSS: Expanded Disability Status Scale.

### Iron rim in paramagnetic lesions imaging and its implications for inflammation

Both histology and MRI studies show alterations in the normal constitutive iron distribution in MS. Iron accumulates in gray matter and should not appear in white matter.

Iron accumulation is cytotoxic, inducing oxidative stress, glutamate toxicity, proinflammatory cytokines increase, and cell repair failure. Iron is a cofactor of enzymes involved in oligodendrocytes and myelin preservation, and may be crucial in remyelination. The extracellular matrix, a key regulator of remyelination, modulates iron availability (122).

In MS, iron-laden astrocytes were scarce in the iron rim and showed weaker iron reactivity than microglia/macrophages (123).

Rim lesions, characterized by a paramagnetic rim in MRI, reflect chronic inflammatory demyelination in MS patients in acute progressive and chronic phases, and in patients with relapse and progressive disease (31).

Consensus is required to define rim lesions and validate them as biomarkers of chronic inflammation in routine MS clinical management (124).

**Figure 4:**
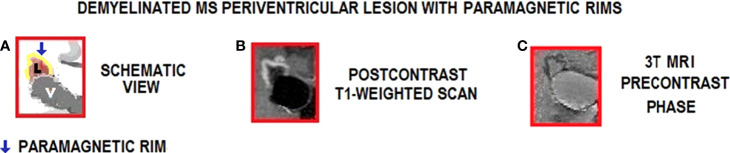
Inflammation in MS periventricular lesions with paramagnetic rims on 3T MRI. Lesion edge shows paramagnetic substances related to inflammation. **(A)** Schematic view of the lesion (pink color) in the periventricular area; **(B)** Active chronic lesion with peripheral gadolinium leak (centripetal pattern) and paramagnetic border (clear zone); **(C)** Active chronic lesion with paramagnetic border (delimited by dark gray border), observed without contrast. Images **(B, C)** show periventricular RRMS lesions with paramagnetic edges (postcontrast and precontrast, respectively, in T1 images); L, Lesion; V, Ventricle.

Rim lesions (rim) in T1-weighted images show a typically complete border of hyperintensity contrasting with the hypointense lesion center and the periphery **(** **Figure 4** **)**.

Inversely, T2-weighted images show a typically complete border of hypointensity compared to the hyperintense lesion center and the periphery that optimally shows fluid and inflammatory changes (125).

Neuroinflammation is always observed with active disease, as in classic active lesions at early stages of the disease and in chronic active lesions affecting white and gray matter related to neurodegeneration upon disease progression.

Iron rim/paramagnetic lesions (IRPL) studies propose annular paramagnetic iron borders around white matter lesions as promising, highly specific MS imaging biomarkers (126).

These images are obtained using a 3T resonator and at a higher resolution with a 7T scanner, which uses a 2-fold magnetic field strength compared with the conventional 3-tesla scanner, though this is not yet available in every medical center (123).

**Figure 5:**
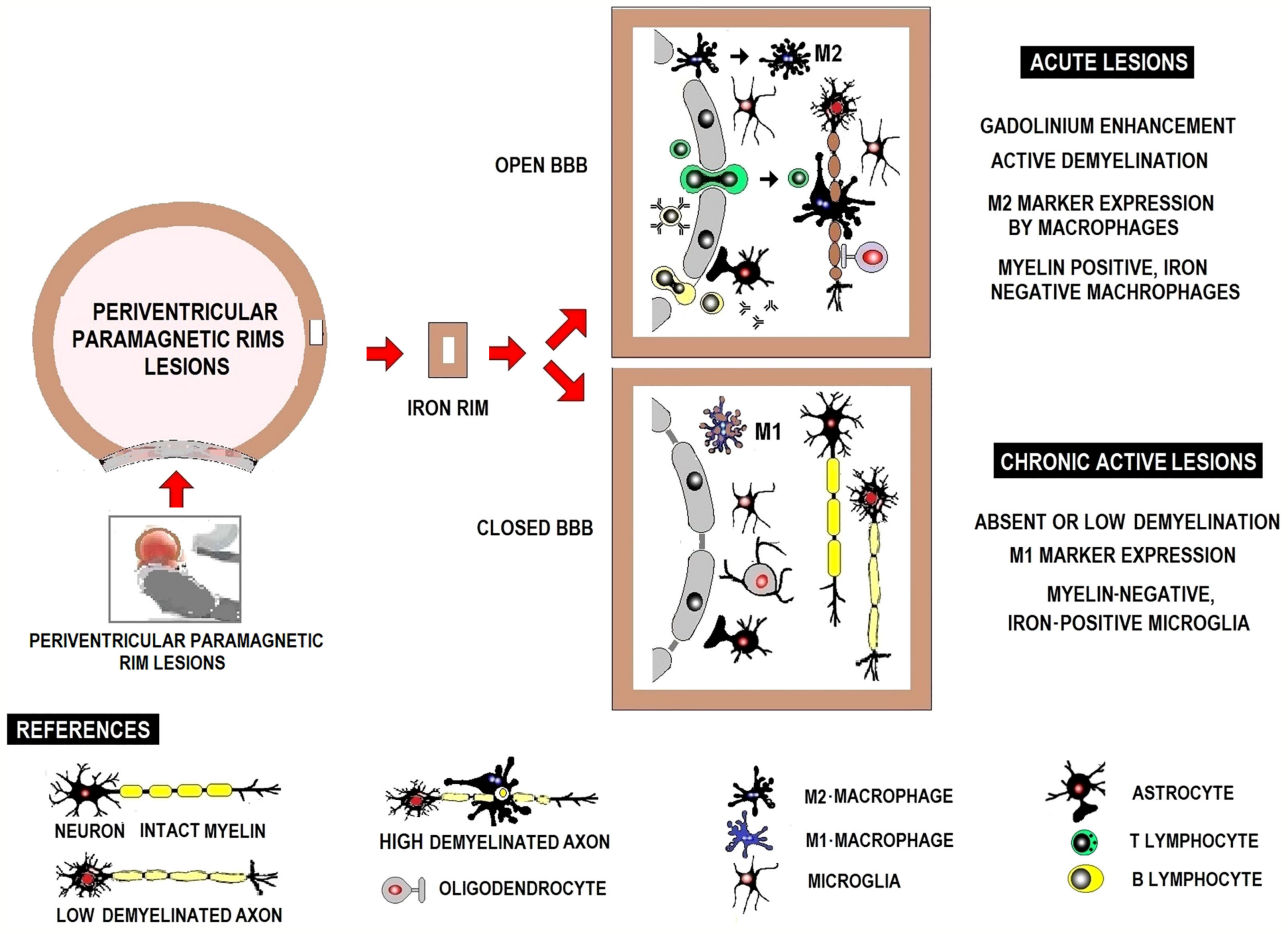
Schematic representation of white matter active acute and chronic lesions and inflammatory infiltrate in periventricular lesions with paramagnetic borders.

Iron, pathologically concentrated in IRPL, is mainly associated with activated microglia/macrophages and less frequently in astrocytes in a pro-inflammatory status. **Figure 5** shows acute and chronic active lesions in white matter and inflammatory infiltrates in periventricular lesions with paramagnetic borders. Acute demyelinating lesions hold activated M2 macrophages. Acute lesions may progress to chronic active lesions with positive iron labeling, particularly in the microglia/macrophages at the edge of the lesion, typically expressing M1-type activation markers (127).

In MS lesions, iron, playing an important role as in any neuroinflammatory disease, can serve as a marker for innate immune system activation. Iron content changes as lesions develop from active demyelination to chronic inflammation and chronic inactivity (12).

Prospective follow-up of “latent” MS active chronic lesions with plain darkened edges, previously detected only at autopsy, suggests that they are related to greater disability at an early age and progressive disease regardless of treatment (13).

### Inflammation, sNFL level, and IRPL in MS

High serum levels of neurofilament light chain (sNFL), an accepted marker of axonal damage, have been associated with *in vivo* IRPL MRI. A multicenter study found higher sNFL levels in MS patients (both RRMS and PMS) without acute disease activity, related to neuroaxonal degeneration that could be detected *in vivo*. Postmortem evaluation showed histological lesions and active chronic lesions with pronounced axonal damage colocalized with chronic inflammatory cells at lesion edges (32). The association between IRPLs and sNFL level was independent of factors affecting sNFL level in previous studies, like age, T2 lesion burden, or DMT treatment.

Most IRPL patients (72%) had sNFL level above the 80th percentile, a pathological threshold according to previous studies (13). The increase in sNFL shows that even a limited amount of ongoing axonal damage at the edge of chronic active/latent lesions, substantially less than in active lesions, can be detected in serum. Focal damage can be detected within a few years by MRI, as some IRPLs are larger and more hypointense on T1 than other MS lesions (123, 128–130).

The number and volume of T2 lesions were unrelated to sNFL level, despite a higher burden of T2 lesions in MRI studies with IRPL ≥ 2 compared with IRPL up to 1 (32). It is essential to identify the subtype of lesions generating neuroaxonal degeneration regardless of clinical and radiological relapses.

IRPL is frequent in RRMS, with nearly 36% of patients presenting at least 2 IRPLs. An inverse association was found between IRPL burden and disease duration, supporting the idea that latent inflammation/demyelination in the early stages of the disease could trigger clinical progression (32, 129, 131). IRPL and clinical disability were associated even without clinical or radiological signs of acute inflammation (32, 128, 132).

Acute demyelinating lesions are gadolinium-enhanced (Gd) in T1-weighted images and contain activated M2-macrophages. Acute lesions may progress to chronic active iron-positive, particularly in the microglia/macrophages at the lesion edge, typically expressing M1- type activation markers, hyperintense, and rarely improving. Silent chronic lesions do not show inflammatory cells.

Currently, 3 Tesla (Discovery 750 -General Electric- with identical platform) and 1.5 Tesla (Hdxt -General Electric- and Achieva -Philips) MRI equipments are available. Clinically approved 7 Tesla (7T) MRI (Magnetom Terra manufactured by Siemens) is rarely accessible. Ultra-high field imaging is particularly advantageous in brain imaging studies. The higher resolution and contrast provided by 7T MRI grant a clear identification of lesions, IRPLs in particular. Yet, there is still time until it is routinely used to detect MS-related chronic inflammatory processes.

### Serum NFL: provisional algorithm for its use as a potential MS biomarker in clinical practice

The so far accumulated evidence on sNFL precludes from generating a reliable algorithm for clinical practice. A reasonably acceptable algorithm may be designed leaning on basic assumptions, and estimated cut-off levels proposed by other authors.

Serum neurofilament light chain (sNFL) poses as an accessible MS biomarker. It is released upon neuroaxonal damage caused by inflammation and its serum level may predict short-term disease progression. Its reliability to reflect long-term evolution is less conclusive. Results from recent worldwide cohort studies are promising, so are clinical trials using sNFL to monitor response to MS treatment.

Clinical scenarios for using sNFL as a potential MS biomarker are: 1) Preclinical MS. As sNFL levels may increase years before the first clinical symptoms appear, they may suggest a risk for a clinical event in patients with RIS. 2) MS diagnosis. Serum NFL level is high in RRMS patients. This might aid in differential diagnosis between CIS and RRMS. 3) MS prognosis. In the short term, high sNFL levels predict the risk of future relapses, new T2 or gadolinium-enhancing lesions, and eventual brain and spinal cord atrophies. Its predictive value in short-term EDSS scores worsening is accepted. 4) MS activity monitoring. Serum NFL level increases upon inflammation and is related to clinical and MRI parameters. Low or steady levels might suggest reduced disease activity, while small increases might anticipate a relapse-free progression. 5) Response to MS treatment and follow-up. A successful response to MS treatment goes with a decrease in sNFL, as shown in clinical trials and population cohorts.

**Figure 6:**
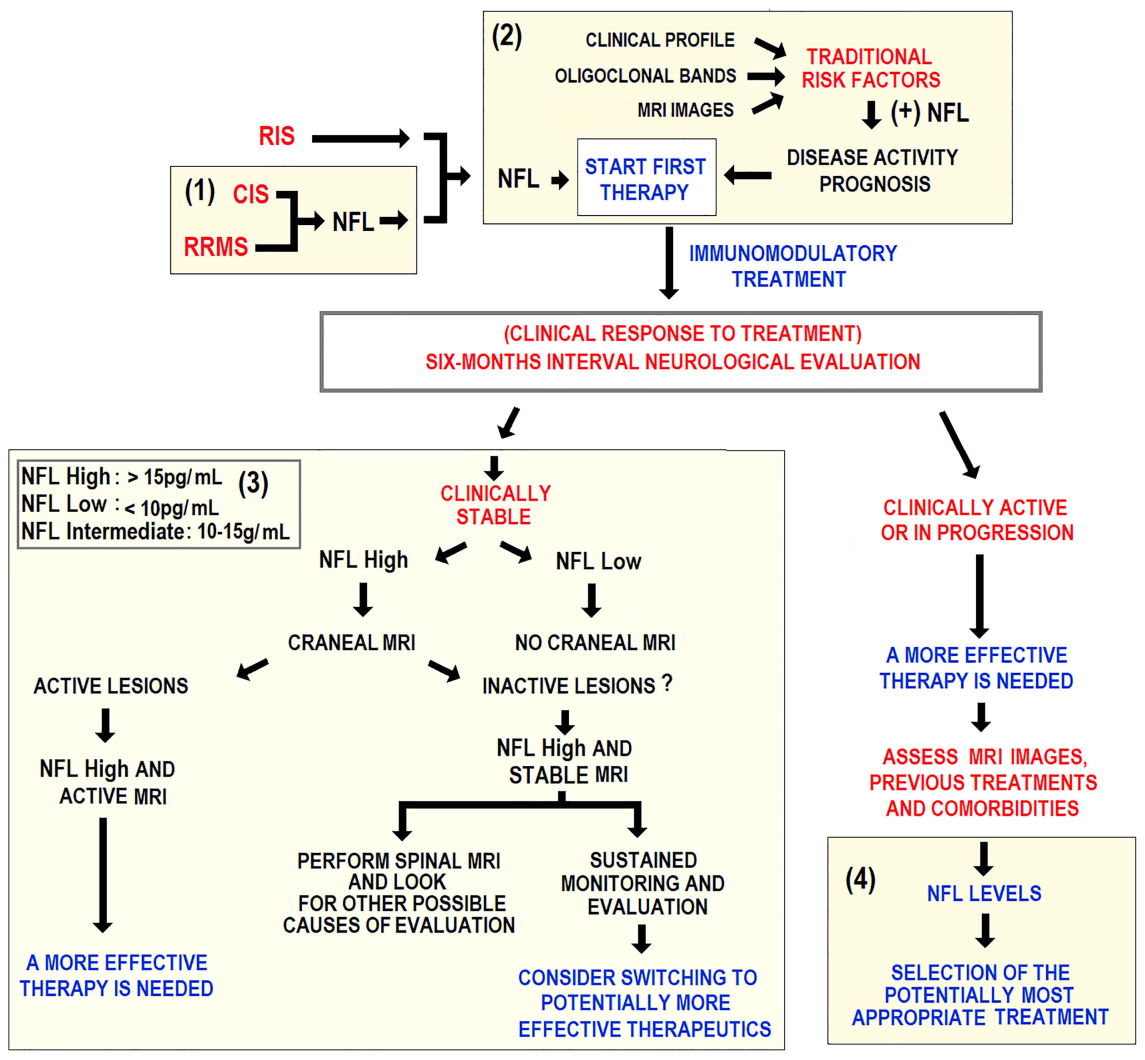
Schematic representation of an algorithm potentially applicable to clinical practice. Longitudinal sNFL measurement is considered for guiding clinical decision-making in RRMS treatment. Yellow fields mark four areas using sNFL as a guidance for decision-making in (1) initial diagnosis, (2) choice of initial treatment, (3) subclinical disease activity assessment, and (4) treatment optimization in clinically active patients.

Provisional clinical algorithms on the routine use of sNFL should be developed for future consensus. These, along with additional biomarkers, clinical data, MRI images should support a customized clinical decision-making in MS patients. An algorithm for decision-making in specific clinical scenarios has been recently proposed (116). These scenarios where sNFL measurement could be indicated include initial diagnosis, treatment choice at first and at any time, and monitoring subclinical disease **(** **Figure 6** **).**

A suitable evaluation algorithm, including sNFL as a biomarker, should distinguish between stabilized patients and those needing therapeutic readjustment. No randomized controlled trial has addressed the question of when to stop treatment in RRMS patients with no evidence of relapse, disability progression, and stable MRI parameters. This concerns patients on the more effective natalizumab or fingolimod therapies due to the well-documented relapse or rebound risk after treatment discontinuation. Provisional clinical algorithms on the routine use of sNFL should be developed for future consensus. These, along with additional biomarkers, clinical data, MRI images should support a customized clinical decision-making in MS patients. An algorithm for decision-making in specific clinical scenarios has been recently proposed (116). These scenarios where sNFL measurement could be indicated include initial diagnosis, treatment choice, and subclinical disease monitoring. The guidelines of the European and American Academy of Neurology (AAN), the algorithm does not recommend treatment interruption in specific patients’ cohorts of patients, while these are a potential scenario to implement sNFL quantification in monitoring and periodic re-evaluations after treatment cessation for clinical reasons.

The suggested stratification in high, intermediate, and low NFL is a rough estimate (116). These partially validated values apply to RRMS patients 18 to ~40-50 years without comorbidities. Beyond this age range, sNFL levels seem to be markedly higher and have been less studied. cMRI= cranial MRI; OCB = oligoclonal bands; RIS = radiologically isolated syndrome, sMRI = spinal cord MRI.

A recent multicenter study tested identical serum samples across 17 different international sites. Excellent inter-assay (56%) and inter-site (59%) coefficients of variation for the most widely used commercial NF-light TM assay were reported (83, 92, 133, 134).

Intra- and inter-assay and batch variability, and between-emerging technical platforms’ variability (e.g., the ELLA system) (111) are still to be addressed. Currently, international efforts are ongoing to standardize sNFL measures.

Basic recommendations for publications quality control in publications include:

Using replicates. Samples and standard solution should be measured by duplicate, at least. The number of samples with repeated measurements due to quality criteria should be reported in the methods section.

Intra-assay precision. The mean coefficient of variation (CV) of duplicate determinations should be reported. Intra-assay CVs below 10% are usually acceptable.

Control samples. Three pre-characterized control samples for low, medium, and high NFL concentrations should be included in each assay to monitor matrix effects and determine between-assay CV. Control samples should ideally proceed from patients and from the same material compartment as the samples (e.g., blood (serum, plasma) or CSF).

Inter-assay precision. Inter-assay CVs should be reported. Values below 10% are usually obtained and may reduce the risk of plaque effects that could be misreported as group effects.

Different batches or trial versions. In theory, lot differences should be negligible. However, caution is advised when using different trial versions. If this is the case, it should be disclosed in the method section and the inter-lot CV should be reported.

Blinding. People performing NFL measurements should be blinded to clinical data.

## Conclusions

Recent years have brought large advances in MS research. We have summarized the knowledge of NFL in MS from laboratory data and imaging to clinical meaning in view of the recent advances in immunopathology. Ultrasensitive digital immunoassay technologies like electrochemiluminescence assay and the single-molecule array have enabled reliable NFL detection in peripheral blood. The evidence supports NFL assay accuracy and reliability as a measure of the typical MS inflammatory and degenerative pathology.

Improved images resolution has made possible characterizing the underlying inflammatory and degenerative milieu in MS, correlating imaging findings with peripheral biomarker levels.

Based on *in vivo* MRI-evidenced IRPLs, chronic inflammatory lesions associated with sNFL are highly frequent in MS patients. IRPL and sNFL show a robust association, independent of other factors, and might be linked to neuroaxonal damage and disability in patients without clinical or radiological signs of acute inflammation. This key concept further supports the role of IRPLs along with sNFL as potential biomarkers for patient stratification and treatment monitoring and design of future clinical trials.

Chronic white matter inflammation is associated with high sNFL levels and disease severity in non-acute MS, suggesting that IRPLs contribute to clinically relevant immunoinflammatory neurodegeneration.

However, the viability of using NFL for MS diagnosis and monitoring is limited due to the lack of disease-specificity. Interpreting NFL results may be misleading because of coexistent neurologic conditions other than MS. Understanding the impact of comorbidities like cerebrovascular disease and metabolic conditions like metabolic syndrome, diabetes, dyslipidemia, and others on sNFL concentration is required to acknowledge NFL as a tool for personalized medicine in MS, especially in progressive MS. Customized monitoring of MS patients should integrate clinical, biological, and imaging data. In addition, normal NFL values across age groups and cut-off values are to be determined. So far, a multicenter analytical assay validation to achieve standardized and reliable measures is missing.

To date, MS diagnosis, treatment, and prognosis rely on neuroimaging and clinical findings. Advances in identifying biomarkers like NFLs and their multidimensional impact foster expectation towards a necessary individualized medicine, imperative in MS. NFL is the biological counterpart of CNS axonal damage and has shown sensitivity to clinical and subclinical changes in disease activity and short-term lesion load. It has performed as an excellent indicator of response to treatment and MS predictor in presymptomatic individuals, granting its use as a clinical trial endpoint. The latest technological breakthroughs and those currently under development broaden access to rapid, low-cost, minimally invasive measurements of circulating NFL levels and facilitate performing extended longitudinal studies. These will eventually aid in determining and validating cut-off values, according to individual characteristics, ongoing treatment and neurological comorbidities. In the near future, we trust that indication to measure peripheral blood NF level be included in clinical best practice guidelines on a routine and longitudinal basis in MS. This will influence clinical decision-making, customizing MS patient management with accurate prognosis and optimized follow-up of newly diagnosed MS patients and those with confirmed MS (97).

The evidence shows that sNFL level is useful for diagnosis, prognosis, and monitoring and makes up a valuable biomarker in MS. Newer assays and techniques for NFL detection in serum samples confirm the usefulness of NFL as a robust biomarker. Further studies should determine reliable cut-off values, leading to more customized medical practice.

## Author contributions

RKF: original idea, updated research, writing original draft; MO-L: writing revision, conceptual, structural, and language editing and proofreading; TK, LU: bibliographic research, writing; MLAB, MH: bibliographic research; FC: supervision, funding acquisition. All authors contributed to the article and approved the submitted version.

## Funding

This work was supported by grants to FC from CONICET (PIP 2016–2022 n. 0779), the University of Buenos Aires (UBACyT 2017–2022), and FONCyT (PICT 0031 2016-2022).

## Conflict of Interest

The authors declare that the research was conducted in the absence of any commercial or financial relationships that could be construed as a potential conflict of interest.

## Publisher’s note

All claims expressed in this article are solely those of the authors and do not necessarily represent those of their affiliated organizations, or those of the publisher, the editors and the reviewers. Any product that may be evaluated in this article, or claim that may be made by its manufacturer, is not guaranteed or endorsed by the publisher.

## Footnotes

1 (*) Rudolph Albert von Kölliker (6 July 1817 – 2 November 1905) was a Swiss anatomist, physiologist, and histologist, grandfather of the first author of this work.

## Abbreviations

AAN, European and American Academy of Neurology; ALS, amyotrophic lateral sclerosis; APC, antigen-presenting cell; bNFL, blood neurofilament light chain; Breg, regulatory B cells; CIS, clinically isolated syndrome; CSF, cerebrospinal fluid; CTL, cytotoxic T lymphocytes; CV, coefficient of variation; DMT, disease-modifying treatment; EAE, experimental autoimmune encephalomyelitis; ECL, electrochemluminescence; EDSS, expanded disability status scale; ELISA, enzyme-linked immunosorbent ultrasensitive assay; Ella, enzyme-linked lectin assay; Gd, gadolinium; IL, interleukin; IRPL, iron rim paramagnetic lesions; MBP, myelin basic protein; MHC, Major histocompatibility complex; MOG, myelin oligodendrocyte glycoprotein; MRI, magnetic resonance imaging; MS, multiple sclerosis; NF, neurofilament; NFH, neurofilament heavy chain; NFL, neurofilament light chain; NFM, neurofilament medium chain; OCB, oligoclonal bands; OFSEP, Observatoire Francais de la Sclerose Plaques; PLP, proteolipid protein; PPMS, primary progressive MS; RIS, Radiologically isolated syndrome; ROS, reactive oxygen species; RRMS, relapsing-remitting MS; Simoa, Single-molecule array; SMSC, Swiss MS cohort; sNFL, serum neurofilament light chain; SPMS, secondary progressive MS; Th, T helper; Th1, T helper type 1; Th2, T helper type 2; Th3, T helper type 3; Th17, T helper type 17; TNF-α, tumor necrosis factor α; Treg, regulatory T cells.
